# Two Cases of Subvesical Bile Duct Injury Detected and Repaired Simultaneously during Laparoscopic Cholecystectomy

**DOI:** 10.1155/2019/3873876

**Published:** 2019-03-26

**Authors:** Atsushi Kohga, Kenji Suzuki, Takuya Okumura, Kiyoshige Yajima, Kimihiro Yamashita, Jun Isogaki, Akihiro Kawabe

**Affiliations:** Division of Surgery, Fujinomiya City General Hospital, Fujinomiya, Japan

## Abstract

**Introduction:**

Subvesical bile duct (SVBD) injury is a secondary major cause of minor bile duct injury after laparoscopic cholecystectomy (LC). However, this injury is usually not recognized intraoperatively, but postoperatively.

**Case Report:**

Case 1: the patient was an 84-year-old female, preoperatively diagnosed with acute cholecystitis. During LC, a tiny hole in the gallbladder fossa from which bile juice oozing was confirmed. Suturing was performed laparoscopically. Case 2: the patient was an 81-year-old male, preoperatively diagnosed with cholelithiasis. Because of a previous history of gastrectomy, laparoscopic adhesiolysis around the gallbladder was performed. During dissection, a small amount of bile was oozing from the surface of the liver adjacent to the gallbladder fossa. Suturing was performed laparoscopically.

**Conclusion:**

If a small amount of bile juice was detected, meticulous observation not only around the cystic duct stump but also the gallbladder fossa should be performed. Simultaneous laparoscopic suturing was feasible, and an ideal procedure against SVBD injury developed during LC.

## 1. Introduction

Bile duct injury is one of the most severe complications after laparoscopic cholecystectomy (LC) [1, 2]. According to a recent report, the incidence of bile duct injury during LC is approximately 0.5% or less [2]. Subvesical bile duct (SVBD) injury is a secondary major cause of minor bile duct injury, while leakage from the cystic duct stump is the most frequent injury [3, 4]. SVBD is a rare anatomical structure which runs in contact with the gallbladder fossa and has a prevalence of 4% or more [5]. SVBD is classified into four types: (1) segmental or sectional SVBD, (2) accessory SVBD, (3) hepaticocholecystic bile duct, and (4) aberrant SVBD [5].

SVBD injuries are often missed intraoperatively because of their small diameter and a lack of the surgeon's understanding. As a result, SVBD injuries are usually detected postoperatively and mainly treated endoscopically, while some severe cases require reoperation [6, 7]. Herein, we show two cases of SVBD injury detected and repaired simultaneously during LC.

## 2. Case Report

**Case 1 .**

**Figure 1:**
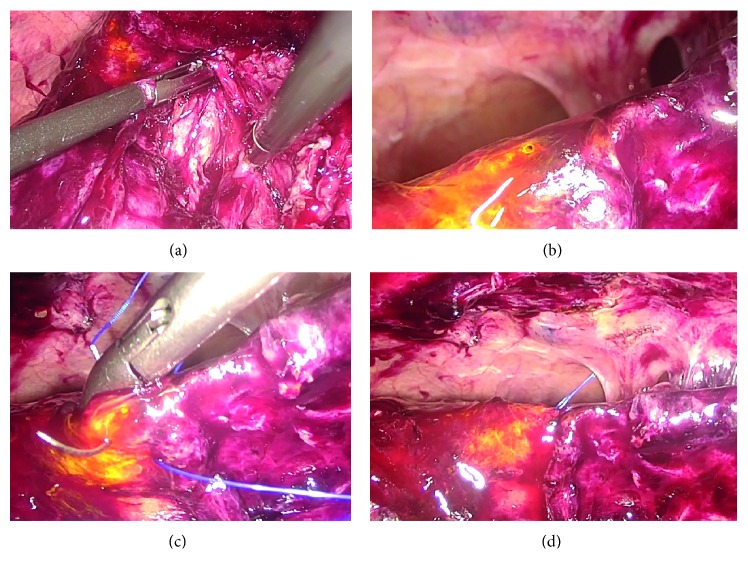
After dissection of the gallbladder from the gallbladder fossa, a small amount of bile leakage was detected in the gallbladder fossa (a). A tiny hole from which bile juice was oozing was confirmed (b). Suturing of the injured SVBD was performed laparoscopically using 3-0 prolene (c). After suturing, the bile leakage stopped (d).

The patient was an 84-year-old female, preoperatively diagnosed with acute cholecystitis. She had a history of undergoing distal gastrectomy with Billroth I reconstruction. On the 3rd day from onset of acute cholecystitis, she underwent LC. Because of the presence of scarring tissue, fundus-down approach was performed. After dissection of the gallbladder from the gallbladder fossa, a small amount of bile leakage was detected at the fundus side of the gallbladder fossa (Figure 1(a)). By scrutinizing laparoscopically, a tiny hole from which bile juice was oozing was confirmed, and we made a diagnosis of SVBD injury (Figure 1(b)). After changing the 5-mm port at the epigastric fossa to a 10-mm port, suturing of the injured SVBD was performed laparoscopically using 3-0 prolene, and the bile leakage was stopped (Figures 1(c) and 1(d)). A closed-suction drain was placed at the subhepatic space. A slight bile leak was detected from the drain on the 1st postoperative day, but it was settled spontaneously on the 2nd postoperative day, and the drain was removed on the 3rd postoperative day. The patient complained of appetite loss, and she was discharged on the 10th postoperative day.

**Case 2 .**

**Figure 2:**
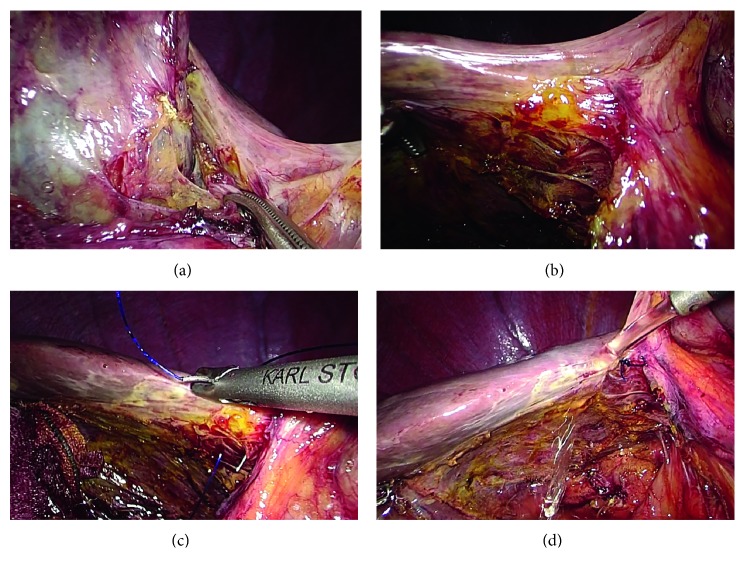
A small amount of bile leakage occurred from the surface of the liver adjacent to the gallbladder fossa (a). After dissection of the gallbladder, the presence of a bile duct injury at the surface of the liver was confirmed (b). Suturing of the injured SVBD was performed laparoscopically (c). After suturing, the bile leakage stopped (d).

The patient was an 81-year-old male, preoperatively diagnosed with cholelithiasis. Because of a previous history of undergoing distal gastrectomy with Billroth I reconstruction, laparoscopic adhesiolysis around the gallbladder was performed followed by cholecystectomy. During adhesiolysis around the gallbladder, a small amount of bile leakage occurred from the surface of the liver adjacent to the gallbladder fossa (Figure 2(a)). After dissection of the gallbladder, the presence of SVBD injury at the surface of the liver was confirmed laparoscopically (Figure 2(b)). Suturing of the injured SVBD was performed laparoscopically, and the bile leakage was stopped (Figures 2(c) and 2(d)). A closed-suction drain was placed in the subhepatic space. Postoperative bile leakage was not detected, and the drain was removed on the 2nd postoperative day. He was discharged without complications on the 4th postoperative day.

**Figure 3:**
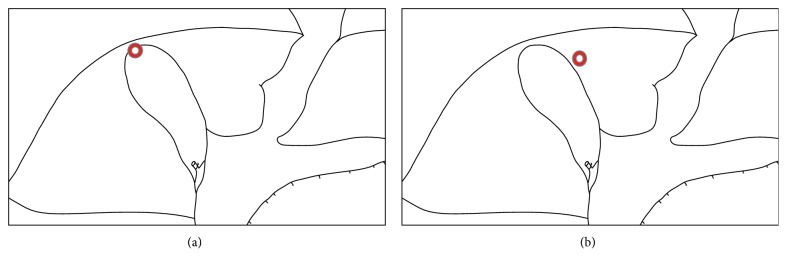
Each circle indicates the location of the injured SVBD in Case 1 (a) and Case 2 (b).

In Case 1 and Case 2, preoperative CT and magnetic resonance cholangiopancreatography (MRCP) images did not indicate the presence of SBVD. In addition, in each case, appearance of the liver was almost normal, without findings of cirrhosis or atrophy. The locations of the injured SVBD are indicated in Figures 3(a) and 3(b), respectively.

## 3. Discussion

We presented two cases of SVBD injury detected and repaired intraoperatively during LC. SVBD injuries are usually detected and diagnosed postoperatively, and intraoperative detection of them is considered rare [5, 6]. The difficulty in intraoperative detection may be derived from the small diameter of the SVBD, decreased bile flow during general anesthesia, and/or the lack of the surgeon's understanding [5, 6]. Only a few cases of SVBD injury detected and treated intraoperatively have been reported previously [6, 8]. Regarding preoperative detection, when reviewing the preoperative MRCP images, we could not recognize the presence of the SVBD in either of the two cases. Although drip-infusion cholangiography CT is thought to have higher sensitivity for detecting the SVBD than MRCP [9, 10], this technique was not performed on our cases. In our cases, meticulous observation around the gallbladder fossa using the laparoscopic imaging system allowed us to successfully detect SVBD injury intraoperatively. Surgeons should consider the presence of SVBD injury if a small amount of bile juice is detected after resection of the gallbladder and should perform meticulous observation not only around the stump of the cystic duct but also around the gallbladder fossa. A recent report advocated the usefulness of intraoperative fluorescent cholangiography for intraoperative detection of the SVBD [11].

According to the classification of the SVBD reported by Shnelldorfer et al., Case 1 is classified as a hepaticocholecystic bile duct, while Case 2 is an accessory SVBD [5]. In Case 1, dissection of the gallbladder from the gallbladder fossa was performed bluntly along the outer side of the subserosal layer because of the presence of the scarring tissue around the fundus side of the gallbladder. The diameter of the injured SVBD was only approximately 2 mm. It seemed that if the dissection was performed using an ultrasonic dissector along the subserosal layer, the SVBD might be sealed without the development of a bile leak. In Case 2, the SVBD injury was developed during adhesiolysis around the gallbladder fossa. This injury was derived from the misperception of the accurate layer for adhesiolysis and cutting into the liver surface adjacent to the gallbladder fossa. We advocate that surgeons should keep in mind that blunt dissection of the gallbladder from the gallbladder fossa along the outer layer of the subserosal layer or cutting into the liver surface adjacent to the gallbladder fossa implies risk for developing SVBD injury.

In addition, our cases demonstrated that simultaneous laparoscopic repair by suturing is a feasible procedure for SVBD injury. According to recently reported cases, SVBD injuries are usually detected postoperatively [12–16]. As a result, endoscopic retrograde cholangiography is usually considered a choice of treatment [6], while there are several cases requiring reoperation including relaparoscopy [6, 7]. According to previous reports, suturing or clipping of the leak site is usually performed during reoperation [7]. Since delayed detection may cause severe conditions including peritonitis [15], intraoperative detection and simultaneous repair is considered an ideal treatment for SVBD injury. On the other hand, there are some limitations regarding laparoscopic suturing. At first, laparoscopic suturing is a skilled procedure and should be performed in expert hands. Second, an excess stitch may result in a development of another injury. Therefore, surgeons should perform intraoperative cholangiography or consider conversion to laparotomy before suturing in the case with a high risk of another injury.

## 4. Conclusions

We reported two cases of SVBD injury detected and repaired during LC. Although detection and ligation of SVBD before injuring them is ideal, surgeons should consider SVBD injury if a small amount of bile juice is detected after dissection of the gallbladder and should perform meticulous observation of the gallbladder fossa before finishing the operation. Simultaneous laparoscopic suturing is a feasible and ideal procedure for SVBD injury developed during LC.
